# Enhancing the drug ontology with semantically-rich representations of National Drug Codes and RxNorm unique concept identifiers

**DOI:** 10.1186/s12859-019-3192-8

**Published:** 2019-12-23

**Authors:** Jonathan P. Bona, Mathias Brochhausen, William R. Hogan

**Affiliations:** 1 Department of Biomedical Informatics, University of Arkansas for Medical Sciences, Little Rock, AR USA; 2 Department of Health Outcomes & Biomedical Informatics, University of Florida, Gainesville, Florida USA

**Keywords:** Drug ontology, Ontology development, National drug codes, Rxcuis, Drug identifiers, Ontological realism

## Abstract

**Background:**

The Drug Ontology (DrOn) is a modular, extensible ontology of drug products, their ingredients, and their biological activity created to enable comparative effectiveness and health services researchers to query National Drug Codes (NDCs) that represent products by ingredient, by molecular disposition, by therapeutic disposition, and by physiological effect (e.g., diuretic). It is based on the RxNorm drug terminology maintained by the U.S. National Library of Medicine, and on the Chemical Entities of Biological Interest ontology. Both national drug codes (NDCs) and RxNorm unique concept identifiers (RXCUIS) can undergo changes over time that can obfuscate their meaning when these identifiers occur in historic data. We present a new approach to modeling these entities within DrOn that will allow users of DrOn working with historic prescription data to more easily and correctly interpret that data.

**Results:**

We have implemented a full accounting of *national drug codes* and *RxNorm unique concept identifiers* as *information content entities*, and of the processes involved in managing their creation and changes. This includes an OWL file that implements and defines the classes necessary to model these entities. A separate file contains an instance-level prototype in OWL that demonstrates the feasibility of this approach to representing NDCs and RXCUIs and the processes of managing them by retrieving and representing several individual NDCs, both active and inactive, and the RXCUIs to which they are connected. We also demonstrate how historic information about these identifiers in DrOn can be easily retrieved using a simple SPARQL query.

**Conclusions:**

An accurate model of how these identifiers operate in reality is a valuable addition to DrOn that enhances its usefulness as a knowledge management resource for working with historic data.

## Background

The Drug Ontology (DrOn) is a modular, extensible ontology of drug products, their ingredients, and their biological activity [1–4]. It was created to enable comparative effectiveness and health services researchers to query National Drug Codes (NDCs) [5] that represent products by ingredient, by molecular disposition (e.g., beta-adrenergic receptor molecule blockade), by therapeutic disposition (e.g., antihypertensive), and by physiological effect (e.g., diuretic). It is based on the RxNorm [6] drug terminology maintained by the U.S. National Library of Medicine (NLM), and on Chemical Entities of Biological Interest (ChEBI) [7].

This paper presents improvements to DrOn in its handling of identifiers used in RxNorm and elsewhere to manage drug information. These improvements include a new model for handling National Drug Codes – which are centrally registered identifiers that denote packaged drug products - as Information Content Entities, and a handling of RxNorm unique concept identifiers (RXCUIs), also as ICEs. Our approach makes it possible to then also model and track changes to these information entities over time. This comprehensive modeling of NDCs and RXCUIs as ICEs, as well as of the entities and processes involved in managing these identifiers, improves DrOn’s representation by bringing it into close correspondence with reality.

With this amendment, DrOn becomes even more useful for prescription data management, especially in those cases where an NDC is used to denote different packaged drug products at different points in time, or where an RXCUI that is now no longer active was used to denote a prescribed product in historic data that needs to be accurately interpreted. Because NDC codes may be re-used [8], without a rich representation of NDC histories, it can be difficult to determine which drug product is denoted by a given NDC in historic records, an issue solved by this proposed amendment to DrOn. RXCUIs are never reused once they are retired but they also require explicit modeling in DrOn to track their evolution over time through their participation in processes such as retirement/deactivation, and various types of remappings [1, 3].

### National Drug Codes

National Drug Codes (NDCs) are numeric codes issued by the US Food and Drug Administration (FDA) and published in a National Drug Code Directory that is updated daily [5]. Each NDC has three segments, which uniquely identify 1) the labeling entity (drug manufacturers, distributors), 2) the drug product, including strength, dose, and formulation, and 3) packaging. Once deactivated, an NDC may be re-used as soon as five years later by the same labeling entity to identify a different product [9]. Though rare, NDC re-use can create difficulties when managing prescription records and other historic data that use NDCs--especially long-term longitudinal databases of pharmacy claims records that span five years or more--because they are not guaranteed to uniquely identify a particular packaged drug product. RxNorm tracks the assignment and deactivation of NDC codes, and this information is made available for each NDC through regular releases of RxNorm. The NLM’s RxNav tools provide the ability to retrieve information about the history of a single NDC using the *ndcstatus* function of the RxNav [10, 11] REST API. As an example, accessing the RxNav REST API gives an XML version of the history for the NDC code 51655072052, shown in Listing 1.

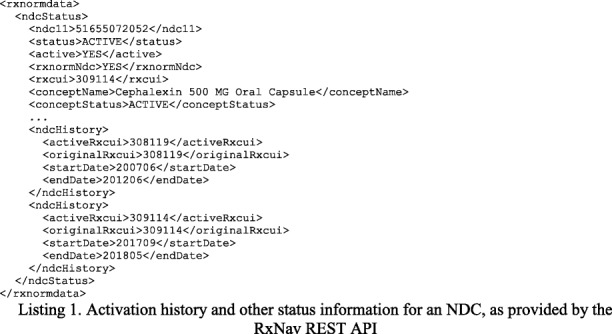



From the perspective of RxNorm, this appears to be a single code that was created once, in 2007, and associated with its first concept/RxCUI at that time (RxCUI 308,119, which identifies the concept *Aminophylline 200 mg Oral Tablet*). The nature of this association is that the NDC code identifies a packaged drug product that contains oral tablets with 200 mg of Aminophylline. Note that there are possibly several other NDC codes that we do not consider here but that also identify packaged drug products containing tablets of the same dose of the same substance, for instance packages that have a different number of tablets than the packaging identified by 51,655,072,052, or packages produced by other manufacturers.

This NDC code 51655072052 was later deactivated, in 2012, ending its association with RxCUI 308,119. Later yet, in 2017, this same NDC code was newly associated with a different RxCUI 309,114, which identifies the concept *Cephalexin 500 MG Oral Capsule*. It is important to note that *Aminophylline Tablets* and *Cephalexin Capsules* have no clear conceptual association but only this accidental connection caused by the reuse of an NDC.

**Fig. 1:**
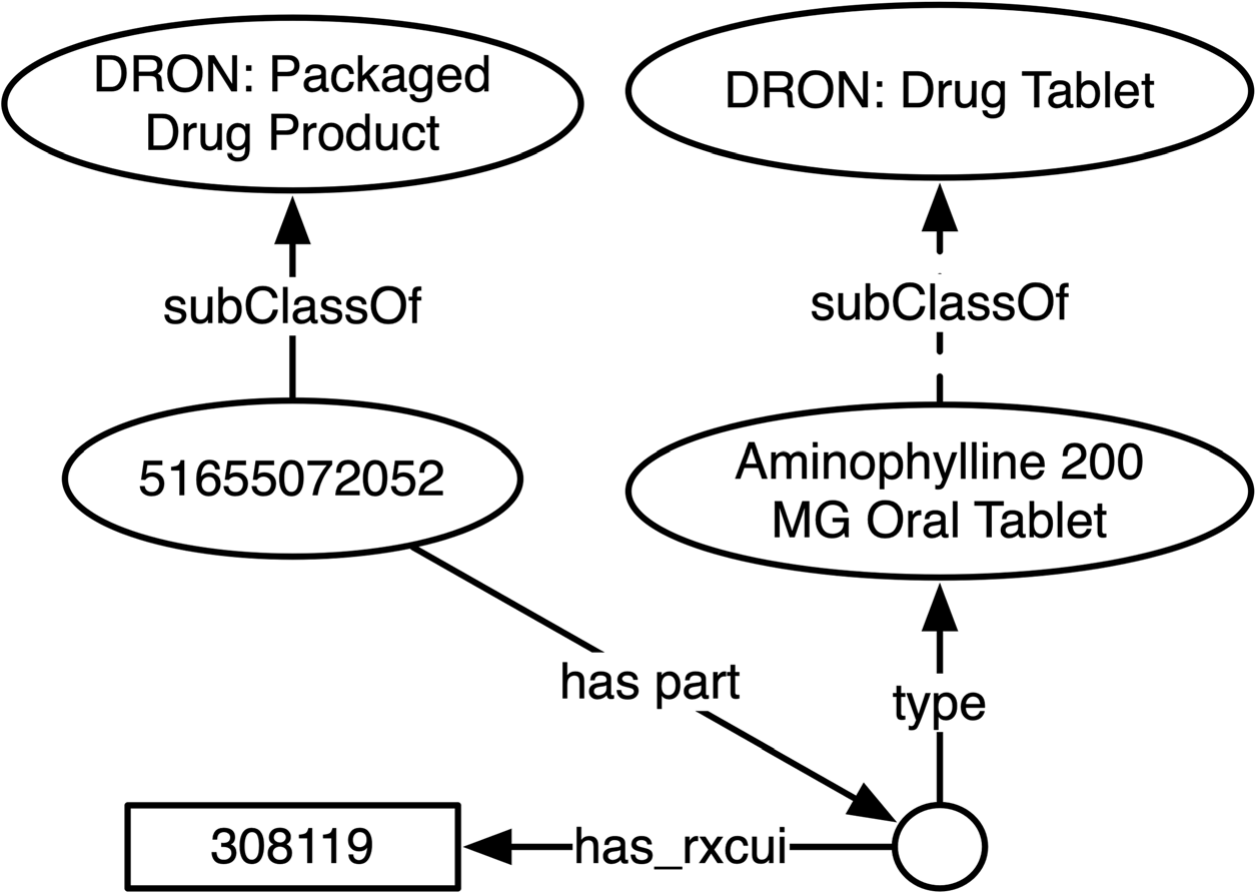
Current representation of a national drug code within DrOn

Currently in DrOn, each NDC is represented as an rdfs:label attached to a class for the corresponding packaged drug product. For example, as shown in Fig. 1, the product with NDC 51655072052, a packaged drug product that includes as part one or more *Aminophylline 200 MG Oral Tablets*, has its own class with 51,655,072,052 as the label. In this scheme, it is not explicitly represented that 51,655,072,052 is an NDC. Further, any historic information about NDCs using the symbol 51,655,072,052 is unavailable to users of DrOn.

In Fig. 1, as in other figures depicting Web Ontology Language (OWL) [12–14] ontology fragments throughout this manuscript we use the following conventions:

- Ovals stand for OWL classes, with the rdfs:label for each class appearing as text in its oval. For example, in Fig. 1, the DrOn class ‘Packaged Drug Product’ is represented by an oval containing the class name.
- Arrows stand for relations. In Fig. 1, as in others, there is a class with a dotted *subClassOf* arrow connecting a specific oral tablet class to the class DRON: drug tablet. These arrows are dotted rather than solid to indicate that the relation in question is not directly asserted in DrOn, but is inferred through the transitivity of the subclass relation.
- Empty circles stand for individuals.
- Rectangles stand for annotation values (aside from rdfs:labels).

To enhance its potential as a tool for managing prescription data, we are adding to DrOn explicit representations of NDCs as Information Content Entities. This representation allows us to account for the creation/assignment, deactivation, and re-use of NDC symbols, and includes temporal information about these processes and links to the correct packaged drug product and drug product classes.

### RxNorm unique concept identifiers

RxNorm is primarily organized around its concepts, identified by RXCUIs (RxNorm unique concept identifiers). These concepts correspond to such entities as drugs, ingredients, dose forms, brand names, etc., and are arranged into a graph that expresses relations among these entities. Concepts are also used to group together synonyms, for instance the different terms that name the same product across drug information sources. For example, the following are all names for an oral tablet consisting of 250 mg of Naproxen [15].

‘Naproxen Tab 250 MG’

‘Naproxen 250mg tablet (product)’

‘NAPROXEN@250 mg@ORAL@TABLET’

‘Naproxen 250 MILLIGRAM In 1 TABLET ORAL TABLET’

‘NAPROXEN 250MG TAB,UD [VA Product]’

RxNorm groups these as synonyms under the same concept, identified by the RXCUI, 198013.

Within RxNorm itself, each of these terms is represented by its own *atom*, and these atoms are grouped together to form concepts. As a realist ontology of drug products, DrOn focuses on representing the products themselves, and information about their ingredients and biological activity, then terminological information such as which string may be used to identify a product in different information sources. Hence, DrOn contains information about RxNorm concepts identifiers, linking these directly to ontology classes that represent the entities that RXCUI concepts correspond to, but does not concern itself with the atoms used to organize synonyms within RxNorm.

Unlike NDCs, RXCUIs are never reused. They can and do, however, undergo other changes. RxNorm tracks concept changes over time with each release. Of course these include the creation of a concept (e.g. for a newly-available drug product), but also concept retirement. RXCUIs may be retired/deactivated for several different reasons, including the removal of a concept that is an error, *merging* two concepts that are discovered to be synonymous (in which case one of the two RXCUIs is retired), or *splitting* a concept into two or more new concepts (in which case the old RXCUI is retired. Once retired an RXCUI is kept in the inactive state through all future releases of RxNorm [16].

**Fig. 2:**
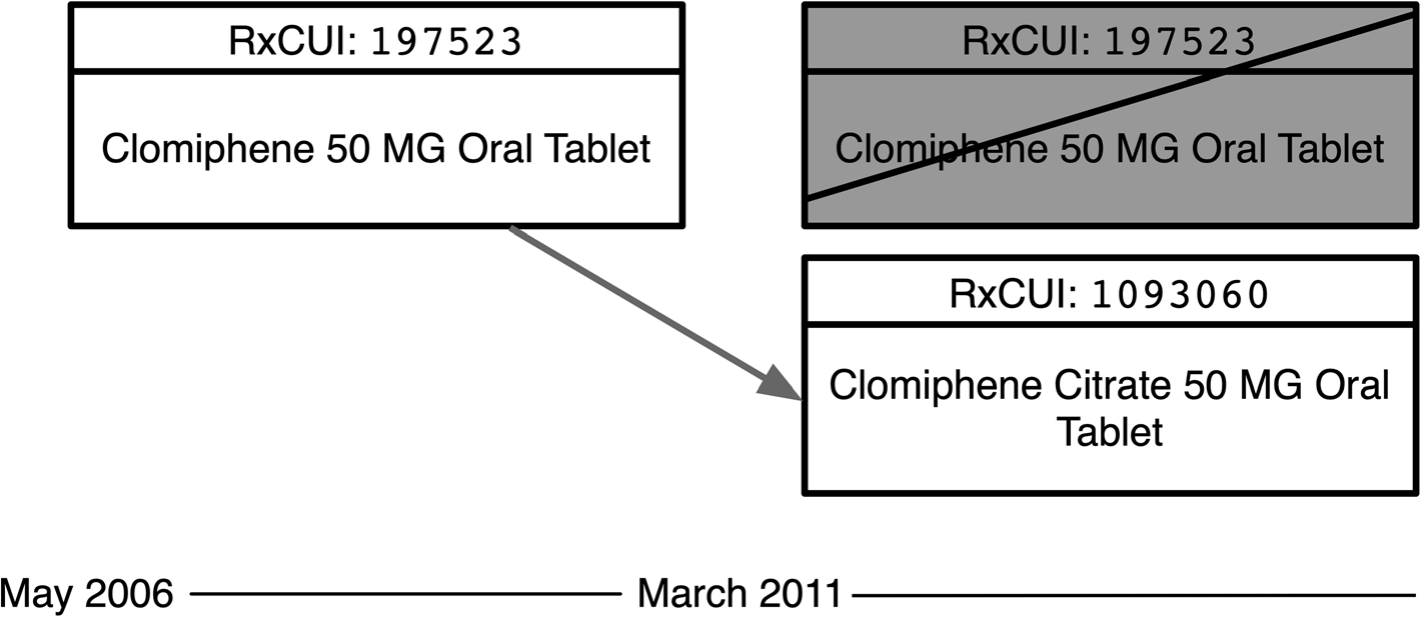
Remapping of RXCUI 197523

Figure 2 shows the **remapping** of RXCUI 197523 (*Clomiphene 50 MG Oral Tablet*), which was created in the May 2006 release of RxNorm, to a new RXCUI 1093060 (*Clomiphene Citrate 50 MG Oral Tablet*) in March 2011. The original RXCUI is retired as part of the remapping.

**Fig. 3:**
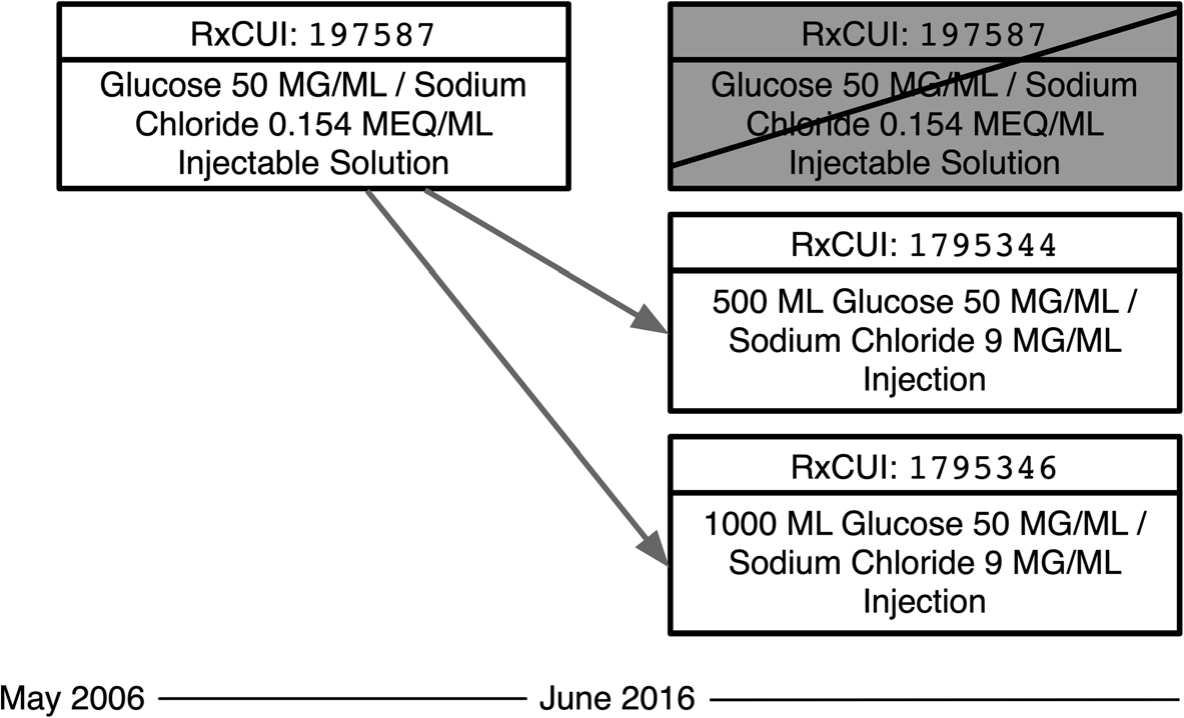
Splitting of RXCUI 197587

Figure 3 shows a **split** in which a single RXCUI is replaced by two others. RXCUI 197587 (*Glucose 50 MG/ML / Sodium Chloride 0.154 MEQ/ML Injectable Solution*) first appears in the May 2006 release of RxNorm. In June 2016 it is split by retiring the original and marking it as replaced by two new RXCUIs: 1795344 (*500 ML Glucose 50 MG/ML / Sodium Chloride 9 MG/ML Injection*) and 1,795,346 (*1000 ML Glucose 50 MG/ML / Sodium Chloride 9 MG/ML Injection*). Note that the distinguishing feature between these two new drug solution concepts is their total volume (500ML vs 1000ML). The original concept does not specify a volume, but only provides concentrations. Conceivably the original concept was used for both 500ML and 1000ML versions before the need to represent this distinction became evident and was included in RxNorm.

Though DrOn’s current build process does use and manage historic information about the provenance of RXCUIs [2] in order to determine which RXCUIs are currently active as of each release, and what entities they currently denote as of that release, a DrOn ontology release that is the output of the DrOn build process does not itself represent the history or provenance of RXCUIs, or make explicit representation of the RXCUIs *as identifiers*. Rather, it simply attaches RXCUIs to the ontology classes that they correspond to using the annotation property ‘has_rxcui,’ as shown in Fig. 1.

This simple representation of RXCUIs allows users of DrOn to easily see the current RXCUI for each class, and to easily query and retrieve current drug information based on RXCUIs, but it is of limited usefulness for working with historic data, for instance to make sense of five year old prescription records stored in an electronic health record system using RXCUIs that were current at the time the data was generated by the system. In this scenario, the current version of DrOn is very useful for answering questions about those entries using RXCUIS that have not changed since the prescriptions were originally written, questions such as: *Which patients were prescribed a product that contains a dose of more than 50 mg of any opioid?* However, for a prescription entry using an RXCUI that has been remapped or otherwise retired DrOn will not be able to provide any information about the drug that was prescribed.

## Methods

This section describes our approach to explicitly representing national drug codes as information content entities and modeling the identifier management processes necessary to accurately represent NDC histories in DrOn, and provides details on our construction of an OWL prototype that demonstrates the approach.

### Explicitly representing national drug codes

In our representation we reuse terms from existing Open Biological and Biomedical Ontology (OBO) Foundry [17] ontologies wherever possible, and add new terms only when necessary. The Information Artifact Ontology (IAO) [18] is an ontology of information entities that provides all representational means (classes, object properties, etc.) for representing identifiers, their parts, and some of the processes of managing them. IAO grew out of the Ontology for Biomedical Investigations (OBI) [19], which covers biological and medical investigations, including many different types of assays that produce or use information artifacts. To represent NDCs and their lifecycles, we add a few classes that extend key classes from IAO and OBI:

- *national drug code*
- *assigning a national drug code*
- *deactivating a national drug code*

These new classes, their connections to existing superclasses in other ontologies, and their definitions appear in Table 1, and are described in more detail below.

**Table 1:**
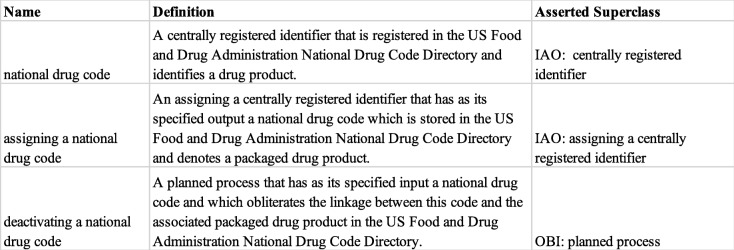
New terms in DrOn used to model NDCs, and their definitions

IAO provides the term ‘*centrally registered identifier*’ (CRID) (http://purl.obolibrary.org/obo/IAO_0000578), defined as “*An information content entity that consists of a CRID symbol and additional information about the CRID registry to which it belongs.*” We are modeling National Drug Codes in DrOn as CRIDs by adding a the class ‘*DRON: national drug code*’ as a new subclass of ‘*IAO: centrally registered identifier*’ (Table 1). Each NDC is represented by an instance of this new class.

Every CRID (including our *national drug code*) has two parts: one denotes the CRID registry, and the other is a CRID symbol. In this case, we use a single instance with the label “*NDC Directory”* to denote the NDC directory. This instance is asserted to *denote* an instance of *‘IAO: centrally registered identifier registry’*. Both of these particular instances are used by each *‘DRON: national drug code’* instance in the ontology. That is, every national drug code instance shares a part with every other. For the NDC CRID symbol we use an instance with the numeric NDC code as its label.

The CRID symbol for each NDC *denotes* the ‘*DRON: packaged drug product*’ to which it has been assigned (and which has the NDC digits printed on its label). Because OWL does not support object property assertions between individuals and classes, we make use of OWL2 *punning* [12] to connect NDC symbols (instances) to the packaged drug products (classes) they are supposed to denote. This is accomplished by having, for each packaged drug product class, an individual that uses the same URI.

### National drug code creation, deactivation, and reuse

Re-use of previously deactivated NDC codes creates potential ambiguities. For instance, consider a use case that we aim to support: determining which drug product was prescribed or administered to a patient based on the occurrence of an NDC code in historic clinical or pharmacy records. Clearly, for records beyond a certain age, this cannot be determined by looking up the NDC alone based on a current version of RxNorm, because it is entirely possible that the NDC denoted a different drug product than it currently denotes at the time this prescription record was added. Our solution to this issue is discussed more below.

We model the process by which an NDC is *created* with ‘*DRON: assigning a national drug code*’, a new direct subclass of ‘*IAO: assigning a centrally-registered identifier*’. To each instance of this assignment process class, we attach a data property assertion indicating *when* it happened.

We also model the process of *deactivating* an NDC. IAO lacks a term for *deactivating/retiring a CRID*. However, it does include ‘*IAO: associating information with a centrally registered identifier in its registry*’, defined as “A planned process in which a CRID registry associates an information content entity with a CRID symbol.” It is not clear whether this term is intended to cover processes such as the deactivation of a national drug code. In this proposal, we add ‘*DRON: deactivating a national drug code*’ as a direct subclass of ‘*OBI: planned process*’. We suggest that IAO would be improved by the addition of one or more terms to support representing deactivation of CRIDs.

**Fig. 4:**
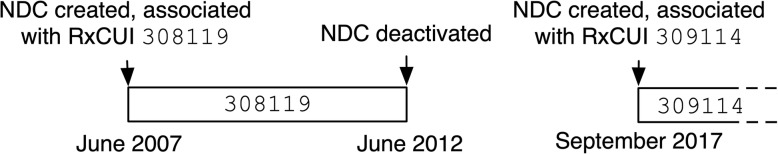
History of NDC codes with digit sequence 51,655,072,052

Because of the ambiguity introduced by NDC reuse, we cannot use single instance of *‘DRON: national drug code*’ (and its CRID symbol part) and assert that it *denotes* multiple different drug products, because this would not allow the retrieval of information about its denotation at different times. A relation like *denotes_during_temporal_region* would support this, but is not feasible to model this in OWL, nor would this necessarily be the most correct way to model it if it were feasible. The reality is that a “reused” NDC is actually a new identifier that happens to look the same because its symbol consists of the same sequence of digits as that of the old NDC, as illustrated in Fig. 4.

This is consistent with the theory of dubbing developed in the Proper Name Ontology [20], and is required by the handling of CRIDs in IAO: the definition of ‘IAO: assigning a CRID’ requires that a new CRID is created as the output of this process:

“*A planned process in which a new CRID is created, associated with an entity, and stored in the CRID registry thereby registering it as being associated with some entity*.”

For these reasons, we treat occurrences of NDCs that use the same sequence of digits at different points in time as separate instances of the national drug code class. That is, to represent the assignment/deactivation history for NDC codes with digit sequence 51,655,072,052, there are two different instances of ‘*DRON: national drug code*’, each the output of a separate ‘*DRON: assigning an NDC identifier*’, one of which occurred in June 2007, and the other in September 2017. The instance whose assignment occurred first denotes the packaged drug product that has as its part a drug tablet that has the RxCUI 308,119 (*Aminophylline 200 MG Oral Tablet*).

**Fig. 5:**
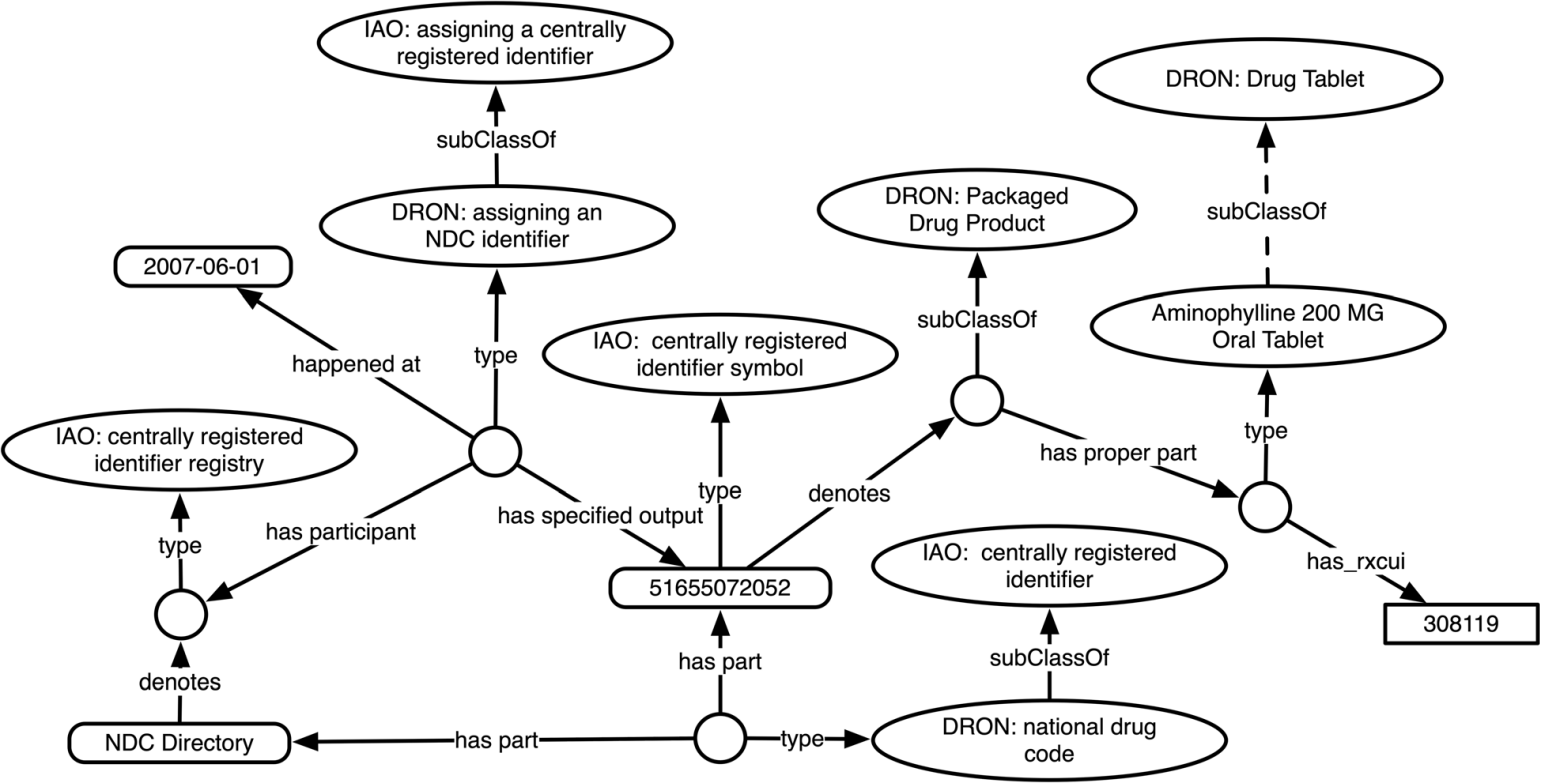
Modeling the processes and other entities involved in creating an NDC

The creation of this NDC, its parts, denotation, and other details are shown Fig. 5. The deactivation of this NDC, which occurred in June 2012, is not pictured there. Neither does Fig. 5 show the creation of the new ‘*DRON: national drug code*’ instance in September 2017 that happened to use the same sequence of digits in its CRID symbol. That new NDC denotes the packaged drug product that has as a part an oral capsule with RxCUI 309,114 (*Cephalexin 500 MG Oral Capsule*).

### Explicitly representing RxNorm unique concept identifiers

In this section we present a model for representing RXCUIs as centrally registered identifiers, and tracking the processes of creating, retiring, and remapping them (including splitting and merging). Our approach to representing RXCUIS and their changes follows the same principles as our representation of NDCs and NDC management processes presented above. We achieve this by using the following new classes:

- *RxNorm unique concept identifier*
- *assigning an RxNorm unique concept identifier*
- *remapping an RXNORM unique concept identifier*
- *retiring an RxNorm unique concept identifier*
- *splitting an RxNorm unique concept identifier*
- *merging RxNorm unique concept identifier*

A full listing of these new classes and their textual and logical definitions appear in Table 2, and their use is described in more detail below.

**Table 2:**
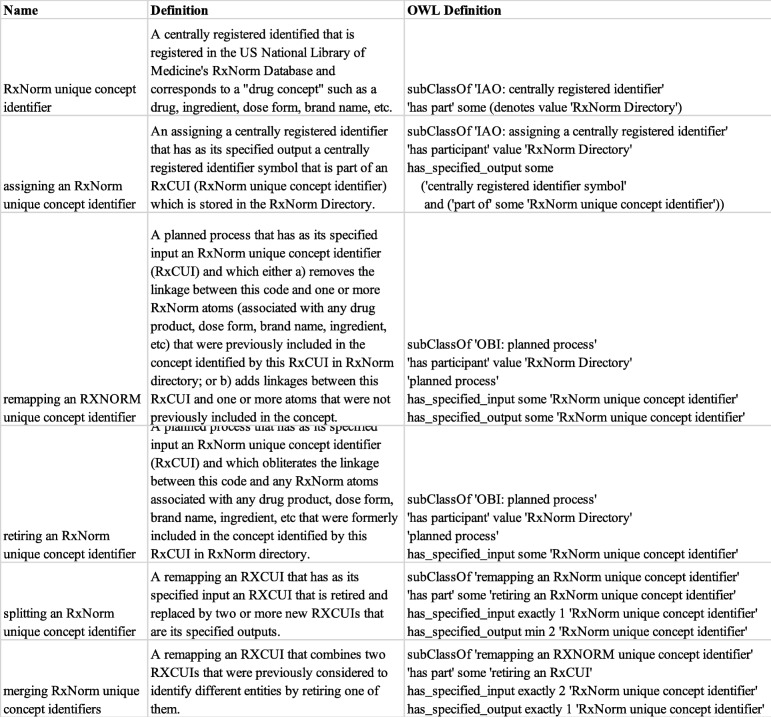
New terms used in DrOn to model RXCUIs, and their definitions

We use the class ‘*RxNorm unique concept identifier*’ (we will refer to this class as *DRON: ‘RXCUI’* for brevity’s sake throughout this section) to represent RXCUIs themselves. Like the class for NDCs, this is a subclass of IAO: ‘*centrally registered identifier*’. Its logical definition requires that each instance of this class has as a part some information content entity that denotes the RxNorm registry.

### Representing RXCUI creation/assignment

Listing 2 shows the status, name, and start and end dates for the concept 308,119 an excerpt retrieved using the RxNav REST API in April 2019 (https://rxnav.nlm.nih.gov/REST/rxcuihistory/concept.xml?rxcui=308119). The startDate field contains the month and year of the release in which this concept first appears in RxNorm, indicating that this concept was assigned in the May 2006 release of RxNorm. Then, as now, it identified the oral tablet ‘Aminophylline 200 MG Oral Tablet’. The endDate contains the month and year of the current release for still-active concepts, or the month and year in which the concept was last active for now-inactive concepts.

**Fig. 6:**
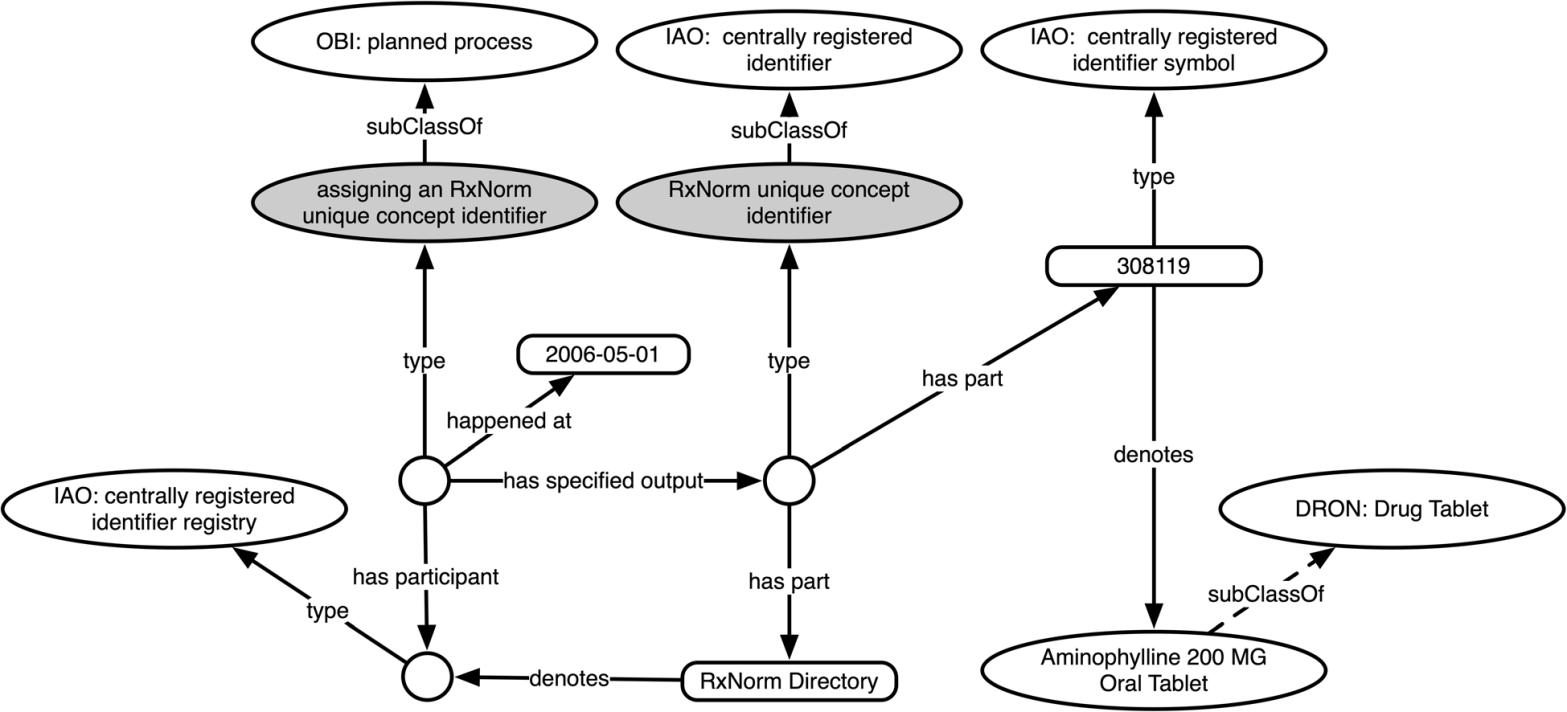
Representing the creation of RXCUI 308119

Each instance of *DRON: ‘RXCUI’* is created by, and linked through its CRID symbol to, an instance of ‘*assigning an RxNorm unique concept identifier*’ (*DRON: ‘assigning an RXUI’* for short). Figure 6 shows as an example of our representation of this process for the concept 308,119. Our representation does not explicitly flag a concept as *Active* or *Retired*: an inactive RXCUI in the ontology will always be linked to an instance of ‘*retiring an RxNorm unique concept identifier*’ that has the concept as its input, as discussed more below.

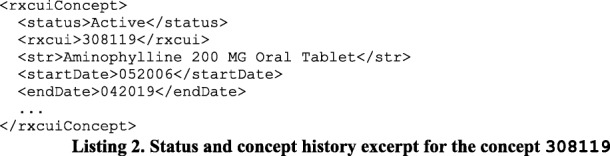



### Remapping / splitting an RXCUI

Recall that, as shown in Fig. 3, one way an RXCUI may be remapped is by “splitting” it into two or more new RXCUIs, for instance because the entities it was previously used to denote are actually better represented by two different concepts. Listing 3 shows how an RXCUI split is indicated in the RxNav API for the concept identified by 197,587. Unlike in the previous example discussed (Listing 2), this concept has the status *Retired*. Another difference is the endDate, which indicates June 2016 rather than the current month (retrieved in April 2019). A remapped RXCUI also has an entry in the currentRxcui field indicating with which RXUI(s) it has been replaced. Here the split is indicated by the presence of two values, 1,795,346 and 1,795,344. To know whether those RXCUIs have been remapped or otherwise retired, or are still active, we would have to separately retrieve status information for each.

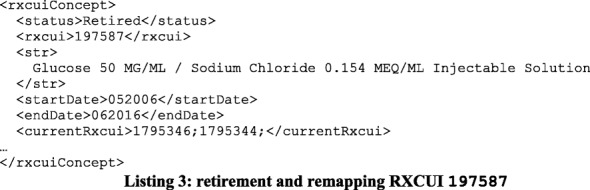



**Fig. 7:**
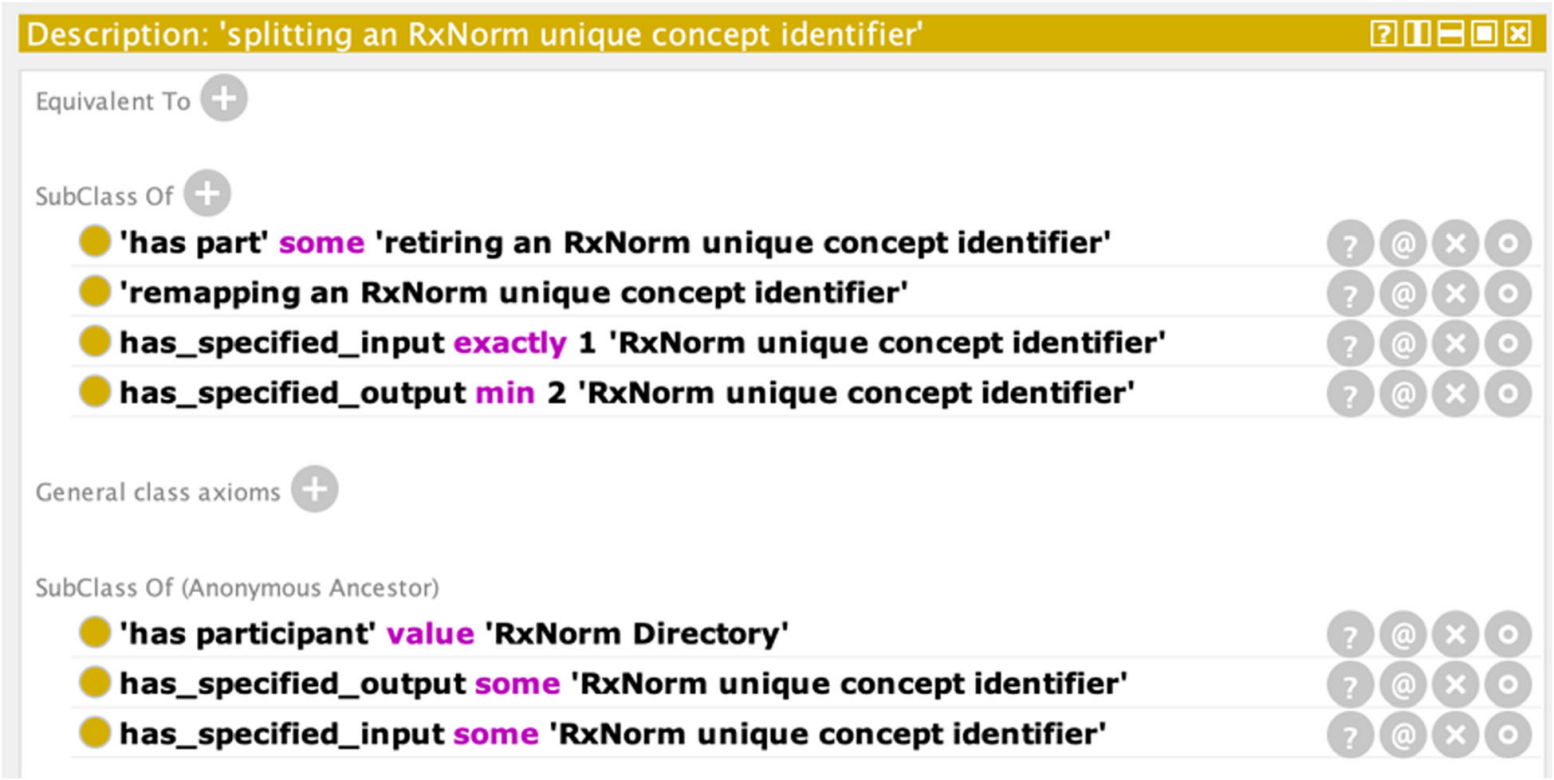
Logical definition for ‘*splitting an RxNorm unique concept identifier’*, as viewed in the Protégé ontology editor

To represent RXCUI remappings that involve splitting an identifier into two or more other identifiers, we define the class ‘***splitting an RxNorm unique concept identifier***’ (***DRON: ‘splitting an RXCUI’*** for short). The axioms defining this class, shown in Fig. 7, specify that this is a remapping process that has one RXCUI as input, has as its part the retirement of an RXCUI, and has two or more RXCUIs as output. The textual definition for this class appears along with the rest in Table 2.

**Fig. 8:**
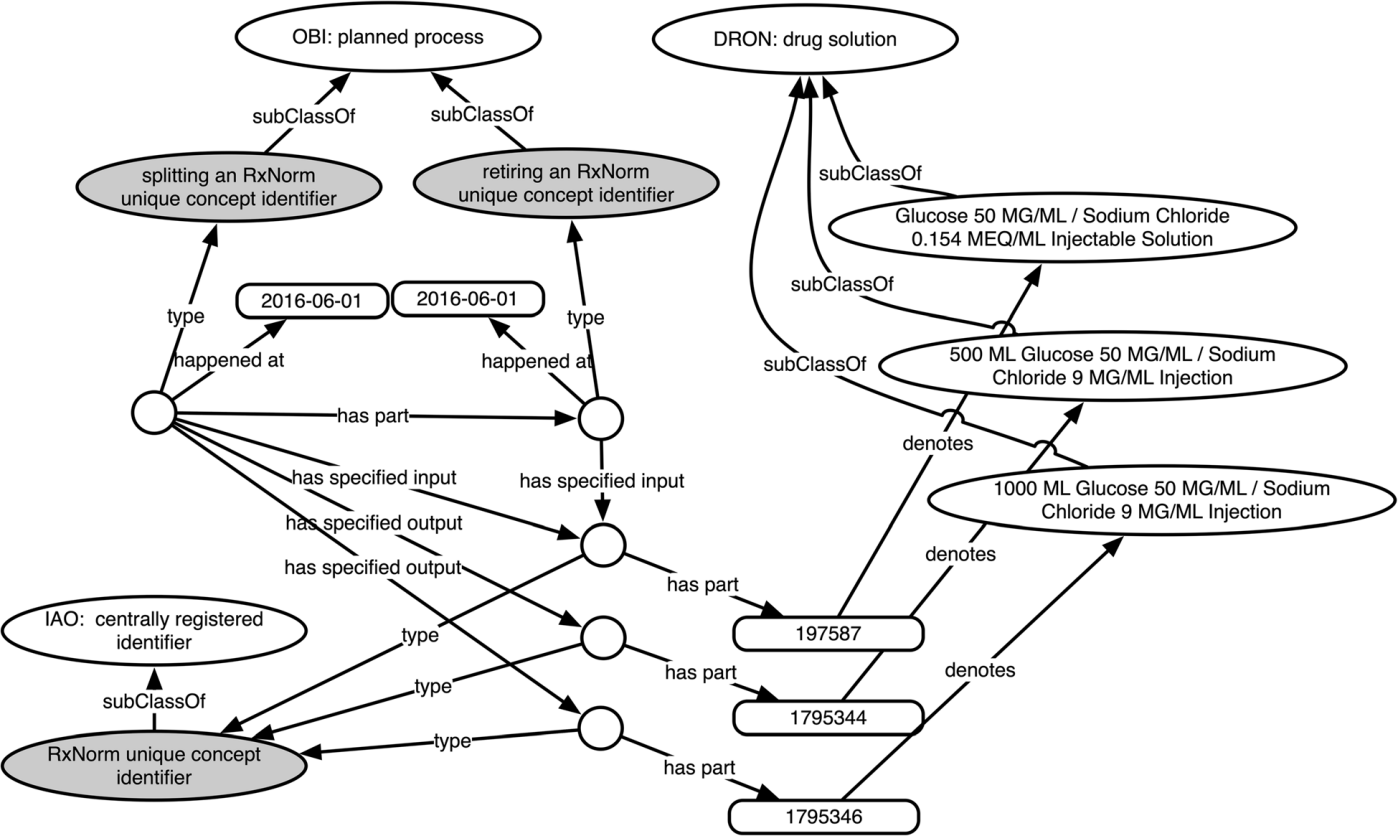
Representing retirement and remapping/splitting RXCUI 197587 in DrOn

Figure 8 shows how we represent within the ontology the replacement of the individual RXCUI 197587 into 1,795,346 and 1,795,344, and that this occurred with the June 2016 release of RxNorm. Note that the original is retired as part of the splitting process. Also as part of this splitting process, the two new RXCUIs are created/assigned, but those processes are omitted from this depiction for legibility’s sake. Also omitted here is the relevant CRID registry, which we have been calling “RxNorm Directory” and its participation in the splitting and retiring processes.

## Results

### OWL implementation

We have implemented these new ontology terms for inclusion in DrOn following the definitions and discussion above. This file was built in Protégé [21], version 5.2. It imports the Information Artifact Ontology and extends relevant classes from both IAO and OBI. The current version of that OWL file is available at http://purl.org/dron/identifiers/dron-identifiers.owl.

### Prototyping instances in OWL

We built a prototype in OWL that demonstrates this approach for a sample of ten arbitrarily-selected NDCs that each has a history of reuse and is present in the most recent release of DrOn. Of these ten, six are currently active and the remaining four have been deactivated, as shown in Table III below.

A set of NDCs with histories of reuse were identified using the RxNav REST API to download NDC statuses as JSON files, and by processing those files with a Python script that examined the length of each NDC’s history to determine whether it had likely been reused. We then examined these ten NDCs to verify that each had been reused. Not all NDCs with multiple history entries have been reused, as discussed in our future work section below.

The OWL draft is built by another Python script that uses the RDFlib library. That script takes as input one or more NDC status JSON files and constructs the representation of each NDC and its history, saving the results in a single OWL file.

**Fig. 9:**
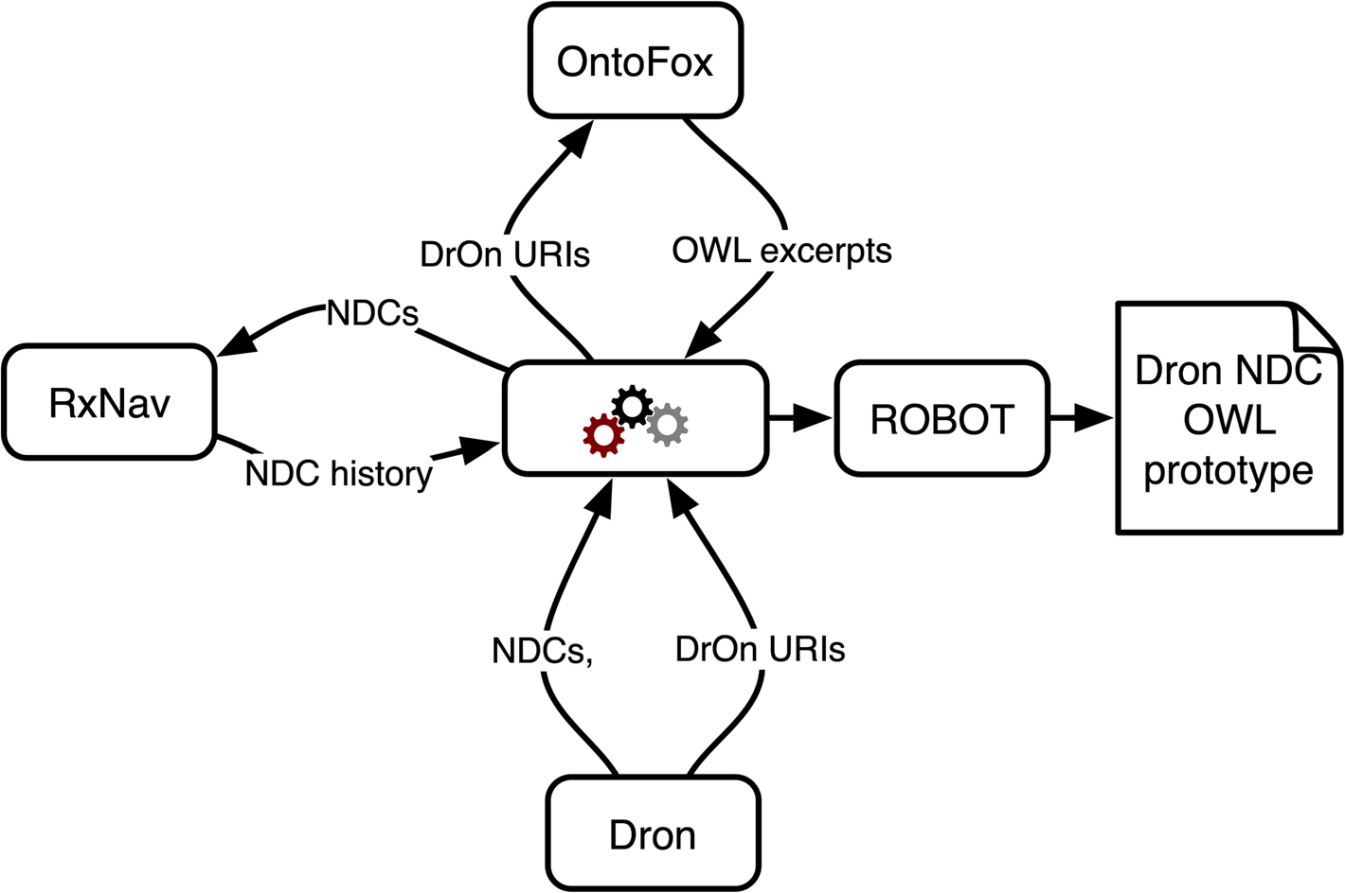
Pipeline for constructing NDC / RXCUI instances prototype in OWL

Building the NDC representation requires linking to existing terms for drugs in DrOn. To identify and extract those terms for use in the prototype, we query a triple store that has DrOn loaded into it, generating and running SPARQL [22] query (using Python’s SPARQLWrapper [23]) for each RxCUI in each NDC’s history to get the corresponding DrOn term. The resulting list of DrOn URIs is then used to automatically generate an Ontofox [24] requests and download a serialization that includes those selected DrOn terms, their label and has_RxCUI annotations, and intermediate terms above them in the hierarchy. It also uses OntoFox to retrieve Basic Formal Ontology (BFO) [25], OBI, and IAO terms. The script invokes the ROBOT csommand line tool [26] to convert between RDF serializations, for instance to convert OntoFox’s RDF/XML output into turtle format for ease of use with RDFLib. This process is shown in Fig. 9.

### Prototype implementation

**Fig. 10:**
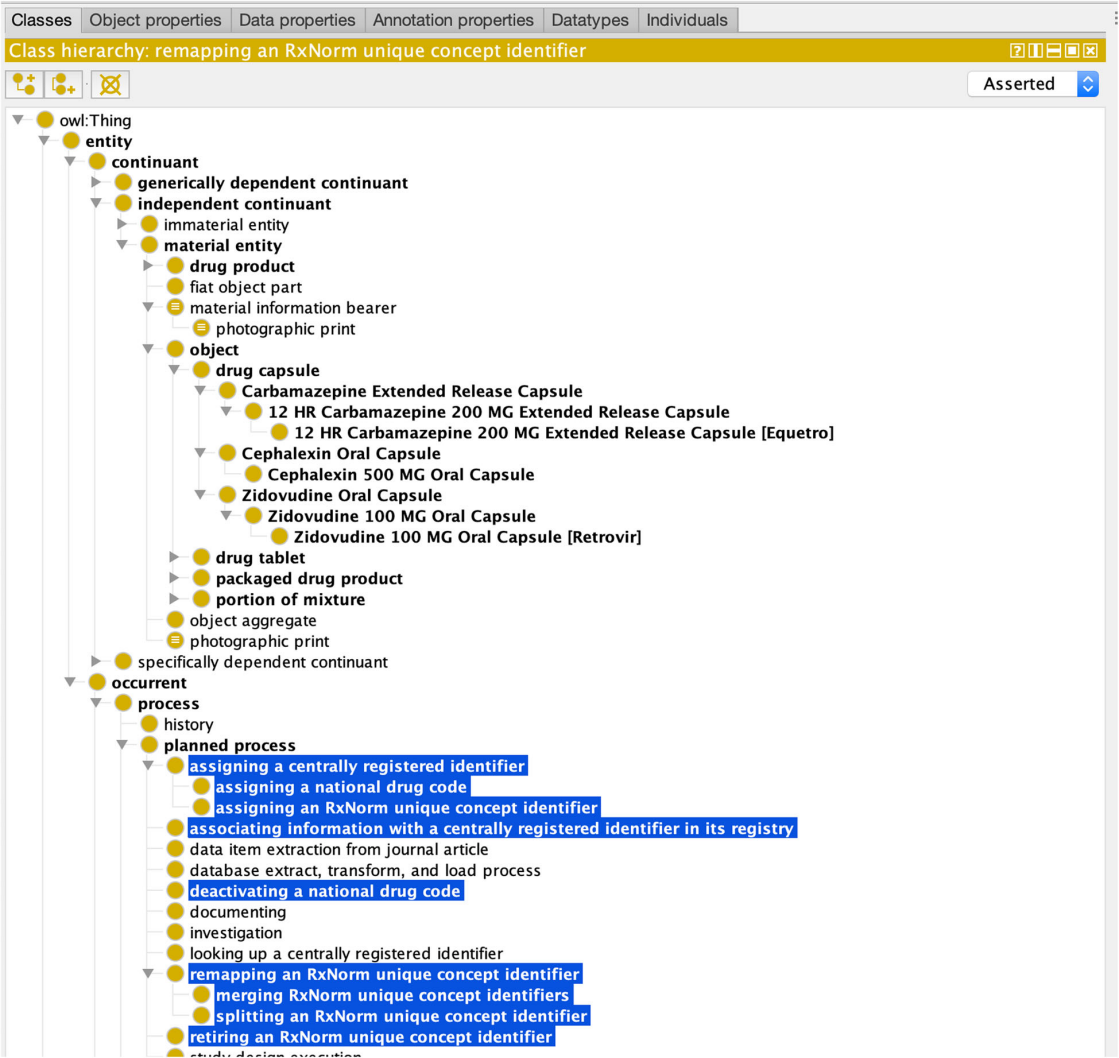
OWL prototype viewed in Protégé, showing major new classes as well as existing DrOn classes to which their instances are linked

Our draft file demonstrating this representation for several instances of NDCs and RXCUIS is available for download in a public BitBucket repository at https://bitbucket.org/jbona/dron-identifiers as a file named ‘ndc_and_rxcui_example.owl’. This file includes DrOn classes for the drug capsules, tablets, etc., that are parts of the packaged drug products that the NDCs denote, as well as the RXCUIs to which those NDCs correspond. Figure 10 shows a selection of these and other OBO ontology classes, and the (highlighted) new classes that we have added to support this work.

This file also contains individuals for the CRIDs and their parts necessary to model the ten NDCs in Table 3, and for the processes involved in their creation/assignments and deactivations. These include:

- 44 instances of ‘*IAO: centrally registered identifier symbol*’
- 22 instances of ‘*DRON: assigning a national drug code*’ and ‘*DRON: national drug code*’.
- 22 instances of *‘DRON: assigning an RxNorm unique concept identifier’* and ‘*DRON: RxNorm unique concept identifier’*.
- 13 instances of ‘*DRON: deactivating a national drug code*’.
- 3 instances of ‘*DRON: retiring an RxNorm unique concept identifier*’.
- Two instances of ‘*IAO: CRID registry*’, and one instance each for the “NDC Directory”, an information content entity denoting the registry that is part of every ‘*DRON: national drug code*’, and an instance that fills the same role for RxNorm concepts.

**Table 3:** Ten NDCs used in prototype

active	obsolete
54,868,026,701	53,002,106,105
17,478,017,101	24,236,032,002
30,698,042,112	52,483,001,400
51,655,072,052	68,115,080,930
00006494301	
47,593,045,731	

As a draft for demonstration purposes, this file does include a few minor peculiarities, including the use of URIs for new terms that start with a temporary URI prefix (http://purl.org/dron/identifiers#) rather than the official DRON prefix, http://purl.obolibrary.org/obo/DRON_. These will be replaced by proper DrOn URIs as this work is integrated with the DrOn build and release processes, and as this representation of NDCs and RXCUIS becomes part of the standard DrOn release.

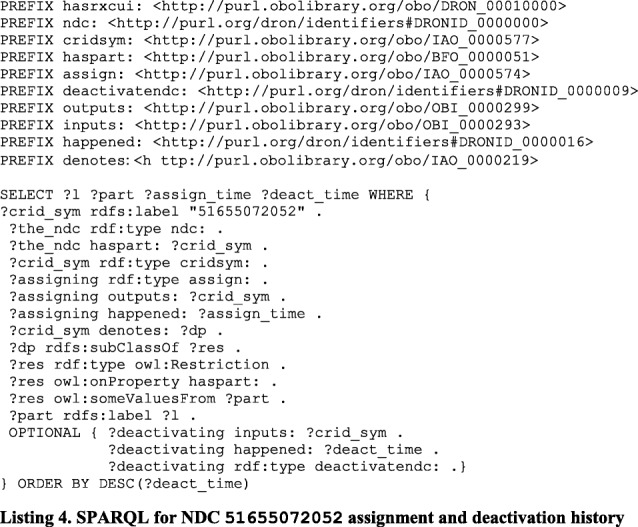



### SPARQL query

The SPARQL query in Listing 4 retrieves information about the assignment and deactivation history for NDCs using the sequence of digits 51,655,072,052. It operates by finding the *CRID symbol* with that label, and finding the *NDC* instance that has this *CRID symbol* as its part. While it is not strictly necessary in this case to find the *NDC*, this ensures that the symbol is part of a CRID of the correct type, which will be necessary in a repository with representations of different types of identifiers. The query finds instances of *assigning a CRID symbol* that have this symbol as their output, and, optionally, instances of *deactivating a CRID symbol* that have the symbol as input, and retrieves the associated timing information for these processes. It also finds the *labeled drug product* that the CRID symbol denotes, and its drug part.

The result of this query is shown in Table 4. Note that this detailed realist representation of NDCs and their management using OWL requires non-trivial SPARQL queries to retrieve even simple information such as the activation and deactivation times for a single NDC. However, once written, such a query can easily be reused to find the same information for other NDCs. This query, for instance, will work to find the history of any NDC in the ontology with only We will provide a selection of useful example queries with the DrOn documentation accompanying these changes when they become part of an upcoming DrOn release.

**Table 4:** NDC 51655072052 history query results

product name	uri	assign_t	deact_t
Aminophylline 200 MG Oral Tablet	DRON_00043869	6/1/07	6/1/12
Cephalexin 500 MG Oral Capsule	DRON_00044386	9/1/17	

## Discussion and future work

We are now working to integrate this work with the official DrOn build process in order to include this explicit representation of identifiers (NDCs and RXCUIs) in future official DrOn releases. Including explicit representations of these entities will noticeably increase the size and complexity of DrOn by adding multiple instances to the ontology file for each identifier, even for those that have not been reused, retired, remapped, etc. Identifiers that have undergone one or more of these changes will require even more instances. It remains to be seen how this addition will impact the usability of DrOn with popular ontology viewing and editing tools such as Protege. Because of its large size and the impact of that on usability, DrOn is currently already distributed as both “full” and “lite” versions, the latter excluding NDCs and other information that may be necessary only for some uses. We will use a similar approach in the distribution of versions of DrOn that include this work on accurately capturing identifiers histories through explicitly representing the identifiers and their management.

We also plan to assess the prevalence of modified identifiers in clinical and pharmacy datasets that we have access to. Such records are at risk of being misinterpreted in analyses that do not take full NDC reuse histories and RXCUI remappings into account.

This assessment will necessarily involve first identifying all NDCs that have been reused. Our preliminary examination of 353,298 NDC codes shows that 71,857 - about 20% - have multiple history entries. Of these multiple history NDCs a, large majority (80%) have only two history items, while about 9400 have three, and 2900 have four. Six NDCs have histories with 30 or more entries. In many cases, these multiple history entries are caused by RxCUI remapping rather than NDC reuse.

## Conclusions

We have developed a full realist ontological accounting of *national drug codes* and *RxNorm unique concept identifiers* as *information content entities*, and of the processes involved in managing these, including assignment and deactivation/retirement processes, and processes involving multiple RXCUIs such as merging or splitting them. We have built an ontology file that implements and defines the classes to represent these entities. We have also built an OWL prototype that demonstrates the feasibility of this approach to representing NDCs and RXCUIs and the processes of managing them by retrieving data for several NDCs, both active and inactive, and the RXCUIs to which they are connected, and representing these instances as individuals in OWL. Finally, we have demonstrated that when this OWL representation is loaded into a triple store database for query and manipulation, historic information about drug identifiers can be easily retrieved using a SPARQL query.

An accurate model of how these identifiers operate in reality is a valuable addition to DrOn that enhances its usefulness as a knowledge management resource, particularly for working with history data. With this addition, DrOn can be used determine what packaged drug product an occurrence of an NDC in a database actually denotes, even for NDCs that are ambiguous in the sense that they are assigned to different products at different points in time. It can also now be used to interpret historic data that refers to RXCUIs that have changed since the point in time that the data was created, for instance to query longitudinal drug prescription records to identify all instances in which a patient was prescribed some opioid analgesic.

## Data Availability

OWL Ontology files are provided via a git repository linked within the manuscript.

## Abbreviations

API: Application Programming Interface
ChEBI: Chemical Entities of Biological Interest
CRID: Centrally Registered Identifier
DrOn: The Drug Ontology
FDA: United States Food and Drug Administration
IAO: Information Artifact Ontology
ICE: Information Content Entity
JSON: JavaScript Object Notation
MG: Milligram
ML: Milliliter
NDC: National Drug Code
NLM: United States National Library of Medicine
OBI: Ontology for Biomedical Investigations
OBO Foundry: Open Biological and Biomedical Ontology Foundry
OWL: Web Ontology Language
REST: Representational State Transfer
RXCUI: RxNorm Concept Unique Identifier
SPARQL: SPARQL Protocol and RDF Query Language
URI: Uniform Resource Identifier
XML: Extensible Markup Language
